# Pregnancy and neonatal outcomes of monozygotic twins resulting from assisted reproductive technology: a 10-year retrospective study

**DOI:** 10.1186/s12958-023-01104-7

**Published:** 2023-06-02

**Authors:** Yubin Li, Qiyuan Chang, Qingyun Mai

**Affiliations:** 1 Reproductive Medicine Center, The First Affiliated Hospital, Sun Yat-sen University, Guangzhou, China; 2 Guangdong Provincial Key Laboratory of Reproductive Medicine, The First Affiliated Hospital, Sun Yat-sen University, Guangzhou, China

**Keywords:** IVF, Intracytoplasmic sperm injection, Preimplantation genetic testing, Testicular sperm aspiration, Monozygotic twin

## Abstract

**Background:**

Monozygotic twins (MZTs) are associated with high risks of maternal and fetal complications. Even with the widely used elective single embryo transfer (SET), the risk of MZTs following assisted reproductive technology (ART) treatments remains. However, most studies of MZTs focused on the relevant etiology, with few studies describing pregnancy and neonatal outcomes.

**Methods:**

This retrospective cohort study included 19,081 SET cycles resulting from in-vitro fertilization (IVF), intracytoplasmic sperm injection (ICSI), preimplantation genetic testing (PGT) and testicular sperm aspiration (TESA) performed between January 2010 and July 2020 in a single university-based center. A total of 187 MZTs were included in this investigation. The main outcome measures were the incidence, pregnancy and neonatal outcomes of MZTs. Multivariate logistic regression analysis was performed to figure out the risk factors for pregnancy loss.

**Results:**

The overall rate of MZTs from ART treatment in SET cycles was 0.98%. No significant difference was found in the incidence of MZTs among the four groups (*p* = 0.259). The live birth rate of MZTs in the ICSI group (88.5%) was significantly more favorable than in the IVF, PGT and TESA groups (60.5%, 77.2% and 80%, respectively). IVF resulted in a significantly increased risk of pregnancy loss (39.4%) and early miscarriage (29.5%) in MZT pregnancies compared to ICSI (11.4%, 8.5%), PGT (22.7%, 16.6%) and TESA (20%, 13.3%). The total rate of twin-to-twin transfusion syndrome (TTTS) in MZTs was 2.7% (5/187); however, the TESA group had the highest rate at 20% and was significantly higher than the PGT group (*p* = 0.005). The four ART groups had no significant effect on the occurrence of congenital abnormalities or other neonatal outcomes in newborns from MZT pregnancies. Multivariate logistic regression analysis revealed that infertility duration, cause of infertility, the total dose of Gn used, history of miscarriages, and the number of miscarriages were not related to the risk of pregnancy loss (*p* > 0.05).

**Conclusions:**

The rate of MZTs was similar among the four ART groups. The pregnancy loss and the early miscarriage rate of MZTs was increased in IVF patients. Neither the cause of infertility nor the history of miscarriage was correlated with the risk of pregnancy loss. MZTs in the TESA group had a higher risk of TTTS, placental effects influenced by sperm and paternally expressed genes may play a role. However, due to the small total number, studies with larger sample sizes are still needed to validate these result. Pregnancy and neonatal outcomes of MZTs after PGT treatment seem to be reassuring but the duration of the study was short, and long-term follow-up of the children is needed.

**Supplementary Information:**

The online version contains supplementary material available at 10.1186/s12958-023-01104-7.

## Background

Multiple pregnancies are considered one of the more serious complications of assisted reproductive technology (ART) techniques [1]. At present, extended culture with embryo selection and elective single embryo transfer (SET) are considered effective methods to reduce the incidence of multiple pregnancies [2, 3]. Nevertheless, elective SET does not prevent concurrent natural conception and embryo splitting, so the risk of multiple pregnancies remains [3].

Monozygotic twins (MZTs), resulting from a zygote split into two embryos, are a special type of multiple pregnancy. The incidence of MZTs following ART treatment is elevated at least 2–3 times compared to spontaneous pregnancy, and approximately 1.5% of all clinical in-vitro fertilization (IVF) pregnancies result in MZTs [4]. Some studies have suggested that this phenomenon may be related to a variety of factors, including ovarian stimulation, stimulation protocol, intracytoplasmic sperm injection (ICSI), embryo cryopreservation, extended culture, oocyte age, and genetic factors [5–7].

Women with MZT pregnancies have a higher risk of maternal and fetal complications than those with singleton or dizygotic pregnancies [8]. During pregnancy, MZTs have increased rates of miscarriage, preterm birth, low birth weight, dysplasia, growth restriction, perinatal morbidity, and mortality in pregnant women [8]. MZTs result from both monochorionic (MC) pregnancies and dichorionic (DC) pregnancies. A study reported that 95% of MZTs following ART treatment were monochorionic pregnancies [9]. Due to MC placentation, women are exposed to peculiar severe complications, such as twin-to-twin transfusion syndrome (TTTS), twin reversed arterial perfusion sequence (TRAP), twin anemia-polycythemia sequence (TAPS), single intrauterine fetal demise (sIUFD), and selective intrauterine growth restriction (sIUGR) [10–12]. Preimplantation genetic testing (PGT) and testicular sperm aspiration (TESA) have been widely used in recent years, providing the possibility of obtaining a biological child in patients with chromosomal abnormalities and azoospermia. However, whether embryo biopsy affects neonatal outcomes remains highly controversial, and few studies have reported neonatal outcomes following TESA in azoospermic men.

Considering the potential risks associated with MZTs, more in-depth studies on the association between ART and MZTs are warranted. Most publications on MZTs after ART treatments have focused on determining its incidence and investigating the associated etiology, with few studies describing the pregnancy and neonatal outcomes [6–8]. In this study, we selected SET cycles that occurred from 2010 to 2020 in our center which resulted in collecting MZT data from 187 cases. We analyzed the potential factors affecting the incidence of MZTs and observed the effects of different ART methods on obstetric and neonatal outcomes of MZT pregnancies to provide evidence and guidance for PGT and TESA treatments and improve patient counseling before the initiation of ART treatments.

## Methods

### Study approval

A retrospective study was conducted on MZT pregnancies obtained by SET using IVF, intracytoplasmic sperm injection (ICSI), preimplantation genetic testing (PGT), and testicular sperm aspiration (TESA) between January 2010 and July 2020 in the Reproductive Medicine Center, The First Affiliated Hospital, Sun Yat-sen University, in Guangzhou, China. The study was approved by the Institutional Ethics Committee ([2021]648). The subjects of this study were mainly from Guangzhou and its surrounding area, Guangdong Province, China.

### Subjects

We divided the patients from the initial cohort of 19,081 SET cycles into four groups. Based on the mode of ART fertility treatment in the medical record system, the groups in this study were defined as follows: (1) IVF, (2) ICSI, (3) PGT, and (4) TESA. If a pregnancy test was positive 12 days after embryo transfer, transvaginal ultrasonography was performed at 2 and 3 weeks, and the number of gestational sacs and embryos were recorded in the medical record system. Monozygosity can only be demonstrated in the case of an SET and, when more than one sac or embryo is shown, the chorionicity/zygosity of the embryo was assessed by ultrasound. Monozygotic twin pregnancies were established according to the SET and the observation of two fetuses on vaginal ultrasonography. In the table of Additional file 2, we divided the patients according to the type (fresh or frozen) and day (D3, D5, D6) of embryos. Detailed data were obtained from medical records within the assisted reproductive medical record system to compare patients in the four groups. Data included maternal age, body mass index (BMI), basal follicle-stimulating hormone (FSH) levels, basal luteinizing hormone (LH) levels, the total dose of gonadotropin used, total days gonadotropin was used, history of miscarriages, number of miscarriages, infertility duration, and cause of infertility.

### Pregnancy outcomes

Pregnancy outcomes included live births, preterm births, full-term births, pregnancy days, caesarean deliveries, pregnancy loss, early miscarriages, late miscarriages, and intrapartum complications. Preterm birth was defined as a baby born after 28 weeks and before 37 weeks of gestation. Full-term birth was defined as a baby born after 37 weeks and before 41 weeks of gestation. Pregnancy loss included miscarriage, stillbirth and genetic terminations. Early miscarriage was defined spontaneous abortion occurring before 12 weeks of gestation, and late miscarriage was defined as occurring after 12 weeks and before 24 weeks of gestation.

### Definition of pregnancy complications

Pregnancy complications were extracted for each pregnancy. Twin-to-twin transfusion syndrome was defined, according to the Eurofetus criteria [13], as polyhydramnios in the recipient (deepest vertical pocket ≥ 8 cm before 20 weeks of gestation or ≥ 10 cm after 20 weeks of gestation) and oligohydramnios in the donor (deepest vertical pocket ≤ 2 cm). Selective intrauterine growth restriction was defined as twins with fetal weight discordance > 25% and the estimated fetal weight of the smaller twin below the 10th percentile on the standardized birth weight chart. Preterm premature rupture of membranes (PPROM) was defined as rupture of membranes before going into labor. Gestational hypertension (GH) is the development of new hypertension in a pregnant woman after 20 weeks of gestation (>140/90 mmHg) without signs of preeclampsia, such as the presence of protein in the urine. Pregnant women were diagnosed with gestational diabetes mellitus (GDM) using the oral 75-g glucose tolerance test (OGTT) at 24 to 28 weeks of gestation. The glucose threshold for the diagnosis of GDM was 5.1 mmol/L for the fast blood test, 10.0 mmol/L at 1 h, and 8.5 mmol/L at 2 h. Anemia during pregnancy was defined as maternal peripheral blood hemoglobin < 110 g/L, and thrombocytopenia was defined as maternal platelet count < 100,000 µ/L. Overt hypothyroidism (OH) was defined as an increase in circulating levels of thyroid-stimulating hormone (TSH) or thyrotropin with a decrease in free thyroxine. The diagnosis of Hashimoto’s thyroiditis (HT), also known as chronic lymphocytic thyroiditis (CLD), is based on the histopathological gold standard of diffuse lymphocytic infiltration with numerous lymphoid follicles and the presence of germinal centers. Intrahepatic cholestasis of pregnancy (ICP) was diagnosed following the complaint of pruritus, a total bile acid level ≥ 10 µmol/L and quick disappearance after delivery.

### Neonatal outcomes

Neonatal outcomes included live births, birth weight, low birth weight, and congenital malformations. Low birth weight was defined was the fetus being < 2500 g. Only major malformations were considered and recorded according to the International Classification of Diseases, tenth revision (ICD-10). Congenital malformations involved in this study included cleft palate, congenital heart disease (CHD), leg malformations, and chromosomal abnormalities. Discordant malformations were defined as a pair of twins with one affected and one unaffected fetus or twin pairs with different malformations. Neonatal outcomes involving congenital malformations were obtained from telephone follow-up.

### Statistical analysis

Statistical analysis was conducted using GraphPad Prism 8 and SPSS 23.0. Comparisons between groups were performed using the Chi-square test or the Fisher’s exact test for categorical data and the ordinary one-way ANOVA for continuous data. Bonferroni correction has been done to test the differences in pregnancy complications and congenital malformations. Multivariate logistic regression analysis was performed to figure out the risk factors for pregnancy loss. *P*-value (2-tailed) < 0.05 was considered statistically significant.

## Results

**Fig. 1:**
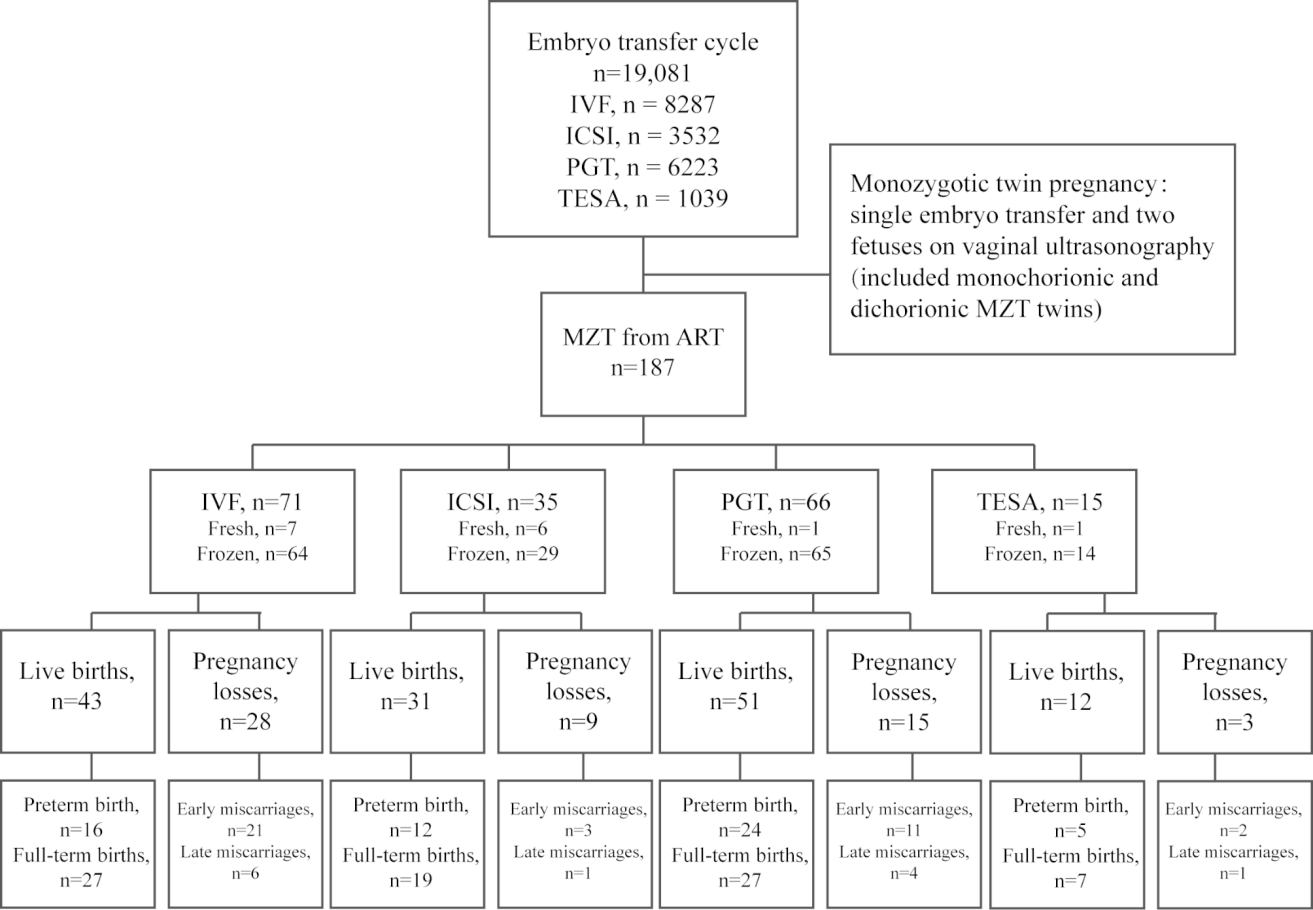
Study population of MZT twins evaluated. IVF, in-vitro fertilization; ICSI, intracytoplasmic sperm injection; PGT, preimplantation genetic testing; TESA, testicular sperm aspiration

From the initial cohort of 19,081 SET cycles, 187 SET cycles were identified as MZTs and were eligible for analysis, exhibiting a rate of 9.8 out of 1000 total cycles. Both fresh and frozen cycles were included for the analysis. There were 71 clinical pregnancies and 43 live births following IVF, 35 clinical pregnancies and 31 live births following ICSI, 66 clinical pregnancies and 51 live births following PGT, and 15 clinical pregnancies and 12 live births following TESA (Fig. 1).

### Baseline characteristics of women among four ART methods

The baseline characteristics of the four groups are detailed in Table 1. Briefly, no statistically significant differences were present among the four groups regarding women’s age, BMI, baseline levels of basal FSH or basal LH, or total duration of ovarian stimulation. The total dose of gonadotropin used showed significant differences among the four groups (*p =* 0.002): 1,940 ± 783.5 for IVF, 1,946 ± 630.2 for ICSI, 2,325 ± 830.2 for PGT, and 1,648 ± 496.4 for TESA. Compared with the other three groups, more women in the PGT group had a history of miscarriage (43/66, 65.1%, *p<*0.001), and the number of miscarriages in this group (1.4 ± 1.3, *p<*0.001) was higher. Female factors (59.2%) were the primary cause of infertility in the IVF group, while male factors (77.1%) in ICSI, and mixed factors (56.1%) in PGT. TESA had the longest duration of infertility (4.60 ± 3.64, *p* < 0.001) and male factors were identified as the main cause (93.3%).

**Table 1:** Baseline characteristics of MZT for different ART methods

Variable	IVF, n = 71	ICSI, n = 35	PGT, n = 66	TESA, n = 15	*P value*
Age (years)	31.9(3.9)	31.8(3.7)	31.6(4.1)	29.1(3.9)	0.085
BMI (kg/m^2^)	21.3(3.2)	20.9(2.0)	21.6(2.7)	19.9(2.1)	0.174
Basal FSH (IU/L)	5.42(1.24)	5.88(1.40)	5.45(1.31)	5.62(0.99)	0.330
Basal LH (IU/L)	4.22(2.93)	3.90(2.52)	3.42(1.70)	4.43(1.94)	0.202
Total dose of Gn used (IU)	1940(783.5)	1946(630.2)	2325(830.2)	1648(496.4)	**0.002**
Total days of Gn used	10.3(2.3)	10.3(1.8)	10.6(2.3)	10.1(2.2)	0.764
History of miscarriages*	26(36.6%)	12(34.2%)	43(65.1%)	1(6.7%)	**< 0.001**
Number of miscarriages	0.6(1.1)	0.4(0.7)	1.4(1.3)	0.1(0.5)	**< 0.001**

Data presented as the mean (SD), data* presented as n (%). BMI, body mass index; FSH, follicle-stimulating hormone; LH, luteinizing hormone; Gn, gonadotropin

### The incidence of MZT among different ART methods

The incidence of MZTs resulting from ART using IVF, ICSI, PGT, and TESA is shown in Additional file 1. The incidence was 0.86% for IVF (0.46% for fresh cycles and 0.95% for frozen cycles), 0.99% for ICSI (1% for fresh cycles and 0.99% for frozen cycles), 1.06% for PGT (0.45% for fresh cycles and 1.08% for frozen cycles), and 1.44% for TESA (0.90% for fresh cycles and 1.51% for frozen cycles). No significant differences were found in the MZT rate among the four groups (*p =* 0.259) regardless of whether the cycles were fresh (*p =* 0.515) or frozen (*p =* 0.430). We also analyzed the MZT rate between fresh and frozen SETs. When the sample included all groups, compared with fresh SETs, the MZT rate was higher in frozen SETs (*p* = 0.048). However, when we analyzed the rate of MZT separately in each group, no significant differences were found between fresh and frozen SETs (*p* = 0.065 for IVF, *p* > 0.999 for ICSI, *p* = 0.731 for PGT and *p* > 0.999 for TESA) as shown in Additional file 1.

### Pregnancy outcomes of MZT among different ART methods

The pregnancy outcomes of MZTs for the four groups are shown in detail in Table 2. The ICSI group had the highest live birth rate (31/35, 88.5%, *p =* 0.012). Live birth rates in the IVF, PGT, and TESA groups were 60.5% (43/71), 77.2% (51/66), and 80% (12/15), respectively. No statistically significant differences were found among the four groups regarding preterm births, full-term births, pregnancy days, caesarean deliveries, spontaneous fetal reduction, and fetal reduction by surgery. The pregnancy days of each group were 248.5 ± 21.9 (35.5 weeks) for IVF, 251.0 ± 15.0 (35.8 weeks) for ICSI, 256.3 ± 15.1 (36.6 weeks) for PGT, and 259.9 ± 18.7 (37.1 weeks) for TESA. Compared with the other three groups, the IVF group had the highest pregnancy loss rate (28/71, 39.4%, *p =* 0.012) and the highest early miscarriage rate (21/71, 29.5%, *p =* 0.049). In the ICSI group, the pregnancy loss rate was 11.4% (4/35). In the PGT and TESA groups, the rates were 22.7% (15/66) and 20% (3/15), respectively. There were no significant differences in the late miscarriage rate among the groups (*p =* 0.743). No significant differences were found involving pregnancy complications among the four groups, except TTTS rates. The total rate of TTTS in MZT pregnancies was 2.7% (5/187); however, the TESA group had the highest rate at 20% and was significantly higher than the PGT group (*p =* 0.005). We also analyzed the pregnancy outcomes among different days of embryo transfer (ET), and no significant differences were found in the rate of live births or pregnancy losses on day 3, day 5, or day 6 of the embryo (*p =* 0.636) as shown in Additional file 2.

**Table 2:** Pregnancy Outcomes of MZT for different ART methods

Pregnancy Outcome	IVF, n = 71	ICSI, n = 35	PGT, n = 66	TESA, n = 15	χ2/R^2^	*P value*				
Live births	43/71(60.5%)	31/35(88.5%)	51/66(77.2%)	12/15(80%)	10.92	**0.012**				
Preterm births	16/71(22.5%)	12/35(34.3%)	24/66(36.4%)	5/15(33.3%)	3.49	0.322				
Full-term births	27/71(38.0%)	19/35(54.3%)	27/66(41.0%)	7/15(46.7%)	2.74	0.434				
Pregnancy days*	248.5(21.9)	251.0(15.0)	256.3(15.1)	259.9(18.7)	0.05	0.090				
Caesarean deliveries	33/43(76.7%)	27/31(87.1%)	48/51(94.1%)	10/12(83.3%)	5.99	0.112				
Spontaneous fetal reduction	12/43(27.9%)	4/31(12.9%)	14/51(27.5%)	5/12(41.7%)	4.47	0.215				
Fetal reduction by surgery	1/43(2.3%)	0/31(0%)	2/51(3.9%)	0/12(0%)	1.68	0.641				
**Pregnancy losses**	28/71(39.4%)	4/35(11.4%)	15/66(22.7%)	3/15(20%)	10.92	**0.012**				
Early miscarriages	21/71(29.5%)	3/35(8.5%)	11/66(16.6%)	2/15(13.3%)	7.86	**0.049**				
Late miscarriages	6/71(8.4%)	1/35(2.8%)	4/66(6.0%)	1/15(6.6%)	1.24	0.743				
**Pregnancy complications** ^**#**^					IVF/ICSI *P value*	IVF/PGT *P value*	IVF/TESA *P value*	ICSI/PGT *P value*	ICSI/TESA *P value*	PGT/TESA *P value*
TTTS	2/71(2.8%)	0/35(0%)	0/66(0%)	3/15(20%)	> 0.999	0.497	0.353	> 0.999	0.023	**0.005**
sIUGR	3/71(4.2%)	1/35(2.9%)	2/66(3.0%)	0/15(0%)	> 0.999	> 0.999	> 0.999	> 0.999	> 0.999	> 0.999
PPROM	7/71(9.9%)	7/35(20%)	6/66(9.0%)	1/15(6.7%)	0.221	> 0.999	> 0.999	0.132	0.407	> 0.999
GH	2/71(2.8%)	2/35(5.7%)	5/66(7.6%)	0/15(0%)	0.597	0.262	> 0.999	> 0.999	> 0.999	0.578
GDM	2/71(2.8%)	4/35(11.4%)	7/66(10.6%)	1/15(6.7%)	0.091	0.088	0.442	> 0.999	> 0.999	> 0.999
OH	1/71(1.4%)	0/35(0%)	1/66(1.5%)	0/15(0%)	> 0.999	> 0.999	> 0.999	> 0.999	> 0.999	> 0.999
HT	0/71(0%)	1/35(2.9%)	0/66(0%)	0/15(0%)	0.330	> 0.999	> 0.999	0.347	> 0.999	> 0.999
ICP	0/71(0%)	1/35(2.9%)	0/66(0%)	0/15(0%)	0.330	> 0.999	> 0.999	0.347	> 0.999	> 0.999
Anemia	1/71(1.4%)	2/35(5.7%)	2/66(3.0%)	0/15(0%)	0.253	0.609	> 0.999	0.608	> 0.999	> 0.999
Fetal distress	0/71(0%)	0/35(0%)	2/66(3.0%)	1/15(6.7%)	> 0.999	0.227	0.174	0.540	0.300	0.468
Thrombocytopenia	1/71(1.4%)	0/35(0%)	0/66(0%)	0/15(0%)	> 0.999	> 0.999	> 0.999	> 0.999	> 0.999	> 0.999
Thromboembolism	0/71(0%)	0/35(0%)	1/66(1.5%)	0/15(0%)	> 0.999	0.482	> 0.999	> 0.999	> 0.999	> 0.999

Data presented as n (%), data* presented as the mean (SD). Data^**#**^ analyzed using the Fisher’s exact test and the Bonferroni correction. TTTS, Twin-to-twin transfusion syndrome; sIUGR, Selective intrauterine growth restriction; PPROM, Preterm premature rupture of membranes; GH, Gestational hypertension; GDM, Gestational diabetes mellitus; OH, Overt hypothyroidism; HT, Hashimoto’s thyroiditis; ICP, Intrahepatic cholestasis of pregnancy

### Neonatal outcomes of MZT among different ART methods

The neonatal outcomes of MZTs for the four groups are detailed in Table 3. There were 72 live birth babies in each group for IVF, 57 for ICSI, 86 for PGT, and 19 for TESA. There was no statistically significant difference in birth weight (*p =* 0.151), low birth weight (*p =* 0.115), or congenital malformations (cleft palate, congenital heart disease, leg malformations, chromosomal abnormalities) among the four ART methods.

**Table 3:** Neonatal outcomes of MZT by different types of ART

Neonatal outcomes	IVF, n = 43	ICSI, n = 31	PGT, n = 51	TESA, n = 12	χ2/R^2^	*P value*				
Live birth babies	72	57	86	19	-	-				
Birth weight(g)*	2329(638.3)	2290(469.9)	2419(518.7)	2593(584.1)	0.02	0.151				
Low weight babies	41/72(56.9%)	36/57(63.2%)	50/86(58.1%)	6/19(31.6%)	5.93	0.115				
**Congenital malformations** ^**#**^					IVF/ICSI *P value*	IVF/PGT *P value*	IVF/TESA *P value*	ICSI/PGT *P value*	ICSI/TESA *P value*	PGT/TESA *P value*
Cleft palate	0/72(0%)	2/57(3.5%)	0/86(0%)	0/19(0%)	0.193	> 0.999	> 0.999	0.157	> 0.999	> 0.999
Congenital heart disease	1/72(1.4%)	0/57(0%)	1/86(1.2%)	1/19(5.3%)	> 0.999	> 0.999	0.376	> 0.999	0.250	0.331
Limb malformations	0/72(0%)	0/57(0%)	1/86(1.2%)	0/19(0%)	> 0.999	> 0.999	> 0.999	> 0.999	> 0.999	> 0.999
Chromosomal abnormalities	0/72(0%)	1/57(1.8%)	0/86(0%)	0/19(0%)	0.442	> 0.999	> 0.999	0.399	> 0.999	> 0.999

Data presented as n (%), data* presented as the mean (SD). Data^**#**^ analyzed using the Fisher’s exact test and the Bonferroni correction

### Multivariate logistic regression analysis for pregnancy loss among different ART methods

Multivariate logistic regression analysis was performed (Table 4) included factors that were significantly different in the baseline characteristics (Table 1): infertility duration, cause of infertility, total dose of Gn used, history of miscarriages, and number of miscarriages. Due to the small sample size of the ICSI and TESA groups (with a history of miscarriages) and their main factor of infertility (male factors), three factors (cause of infertility, history of miscarriages, and number of miscarriages) could not be included in the model in these two groups. However, the analysis revealed that all these factors were not related to the risk of pregnancy loss (*p* > 0.05) in four groups.

**Table 4:** Multivariate logistic regression analysis for pregnancy loss

	B	SE	*P*-value	Odds ratio	95% CI of odds ratio
**IVF**					
Infertility duration	0.105	0.117	0.372	1.11	0.883 ~ 1.396
Cause of infertility	-0.367	0.32	0.251	0.693	0.370 ~ 1.297
Total dose of Gn used	0	0	0.765	1	0.999 ~ 1.001
History of miscarriages	0.525	0.973	0.589	1.69	0.251 ~ 11.369
Number of miscarriages	-0.552	0.584	0.344	0.576	0.183 ~ 1.808
**ICSI**					
Infertility duration	-0.849	0.624	0.174	0.428	0.126 ~ 1.455
Total dose of Gn used	-0.001	0.001	0.566	0.999	0.997 ~ 1.002
**PGT**					
Infertility duration	0.086	0.131	0.513	1.09	0.843 ~ 1.409
Cause of infertility	0.137	0.355	0.7	1.147	0.572 ~ 2.302
Total dose of Gn used	-0	0	0.943	1	0.999 ~ 1.001
History of miscarriages	-0.252	0.999	0.801	0.777	0.110 ~ 5.506
Number of miscarriages	0.395	0.325	0.224	1.485	0.785 ~ 2.808
**TESA**					
Infertility duration	0.195	0.247	0.428	1.216	0.750 ~ 1.971
Total dose of Gn used	0.001	0.001	0.509	1.001	0.998 ~ 1.004

## Discussion

In this 10-year, single-center, retrospective analysis of MZTs after assisted reproduction, we compared the incidence of MZTs among different ART methods and between fresh or frozen transplantation, and we also evaluated and analyzed the effect of four ART methods on obstetric and neonatal outcomes of MZT pregnancies.

Previous studies have suggested that the incidence of MZTs may be associated with fresh or frozen cycles as well as the in vitro culture of embryos [14]. The incidence of MZTs using ART to achieve pregnancy significantly increased compared to MZTs resulting from spontaneous pregnancy [15–17]. In the present study, the overall incidence of MZTs was 0.98% which is consistent with previous studies [15, 18, 19]. Some studies have suggested that embryo processing, such as embryo biopsy, may affect the incidence of MZT pregnancy [17, 20–22]. However, this phenomenon was not observed in this study. Our study with a large amount of data showed that there was no statistically significant difference in the incidence of MZTs among different ART treatments, and the results suggest that the micromanipulation of sperm and embryos involved in ICSI, PGT, and TESA in our laboratory had no significant effect compared to IVF on the incidence of MZT. This conclusion was consistent with studies by Sobek et al. and Dallagiovanna et al. who found that embryo-related micromanipulation techniques did not increase the risk of MZT incidence [8, 21]. In addition, our data showed that frozen SETs had a higher risk of MZT than fresh SETs when we analyzed the total MZT rate, as reported in the previous study [23]. However, when we analyzed the incidence of MZTs in each group, there was no statistically significant difference between fresh and frozen SETs. This discrepancy may be due to the insufficient number of MZTs in each group. Some studies have suggested that the increased MZT rate when undergoing frozen ET may be associated with zona pellucida hardening [7, 24]. In contrast, the study by Nakasuji et al. suggested that embryo cryopreservation did not influence the MZT rate [25]. The association between the MZT rate and frozen ET remains controversial and needs further investigation.

Twin pregnancies were associated with several obstetric complications, some of which led to serious perinatal consequences [26–29]. Perinatal mortality in twin pregnancies can be up to 6 times higher than in singleton pregnancies, which is mainly due to the higher rates of preterm birth and fetal growth restriction in twin pregnancies [30, 31]. Qin et al. showed that multiple pregnancies produced by ART were associated with an increased risk of pregnancy complications and adverse pregnancy outcomes compared with spontaneous conception [32]. The study by Hansen et al. demonstrated that ART infants have a higher risk of birth defects and suggested that further studies are needed to examine the risk for important subgroups of ART exposure [33]. The wide use of PGT worldwide raises a concern about the potential risks of embryo biopsy. Trophectoderm (TE) biopsy removes cells that are destined to form the placenta, leading to an increased risk of pathological placentation in PGT [34]. A large study examining maternal and neonatal outcomes reported a statistically significant three-fold increase in the risk of preeclampsia associated with TE biopsy [35]. In patients with azoospermia, pregnancy can be achieved through TESA using sperm retrieved from the testis instead of ejaculated sperm. However, Jeffrey et al. demonstrated that the growth trajectory of the placenta was changed in pregnancies conceived after TESE-ICSI, and suggested that the origin of sperm may affect placental and fetal development [36]. Ghazzawi et al. reported that the miscarriage rate in TESA-ICSI cycles was significantly higher than ICSI with ejaculated sperm. Another concern in children born after TESA with the use of testicular sperm is the potential risk of malformation [37]. Nevertheless, there has been very little examination of the maternal and neonatal outcomes for multiple pregnancies achieved via PGT and TESA. Two studies with large sample sizes have investigated pregnancy and neonatal outcomes in 242 twin pregnancies after PGT and 176 twin pregnancies after TESA; unfortunately, neither study analyzed MZT independently [38, 39]. In this study, we added the PGT and TESA groups and analyzed the pregnancy and neonatal outcomes of MZT separately. To our knowledge, this study is one of only a few, and the largest to date, that provide detailed data on pregnancy and neonatal outcomes of MZT after PGT and TESA compared with IVF and ICSI.

Pregnancy loss (PL) significantly threatens the rate of live birth delivery, which includes miscarriage, stillbirth, and genetic terminations [40]. Previous studies showed that patients conceiving through ART treatment were confronted with a higher risk of PL than women conceiving naturally, and the ICSI group has similar obstetric outcomes to the IVF group [41, 42]. In contradiction to earlier findings, our data surprisingly found that IVF had a higher risk of PL and early miscarriage for MZTs. The other three ART groups (ICSI, PGT, TESA) all included ICSI processing and had lower PL rates, pointing to the likelihood that ICSI operations may not be detrimental to MZT obstetric outcomes. Although the miscarriage rate was high, the probability was consistent with the previous study, which documented a 50% miscarriage rate in the MC pregnancy group and a 10% miscarriage rate in the DC pregnancy group [43]. As for ART population, the increased potential risk of miscarriage had been associated with some potential factors specific to women with infertility. The duration of infertility can be used as a good indicator to predict the pregnancy outcome of infertile patients [44]. The quality of oocytes and embryos in women with a long duration of infertility may decline, leading to the occurrence of early abortion. Some female factors may also affect embryo implantation and development, leading to early PL [45, 46]. The dosage of Gn in ART cycles is related to many factors such as patient age and ovarian responsiveness, and high total Gn dose likely exerts a negative impact on the endometrium and embryo [47]. In addition, a previous study has found associations of miscarriage history with infertility, subsequent miscarriage, and poor obstetric outcomes [48]. Unfortunately, our logistic regression results found no association between these factors and PL, which was partially consistent with previous study [49]. One possible explanation is that the small number of MZT patients in our study limited the power of conclusions. Embryo-related risk factors of IVF patients also affect the occurrence of early miscarriage. The high rate of chromosome aneuploidy is another major cause of PL and miscarriages in IVF [50, 51]. However, the retrospective nature of this study does not allow us to know whether miscarriage in the IVF group was due to aneuploid embryos. Considering the physical and emotional trauma associated with PL, embryo-related risk factors and other unknown barriers need further prospective studies with larger sample sizes.

The rate of preterm births in MZTs reported in this study was similar to that found in other studies, ranging from 31 to 44% [29, 52, 53]. The average number of gestation weeks of the four groups was consistent with previous studies; on average, twins delivered at 35.3 weeks of gestation [54]. Our study also showed that MZT pregnancies after ART treatments had a high rate of caesarean deliveries, which was compatible with other studies [38, 55]. Twins conceived after ART treatment were more likely to be delivered by caesarean delivery, and these differences may be related to multiple factors, including the history of caesarean deliveries, maternal older age, and prolificacy. The caesarean delivery rate in China has been very high; social factors may be one of the reasons for this difference [56]. Previous studies reported that the prevalence of vanishing twins was as high as 10–40% [57, 58]. Furthermore, compared with naturally pregnant women, the prevalence was higher in twin pregnancies conceived after IVF/ICSI procedures [58–60]. In the present study, the rates of spontaneous fetal reduction were consistent with previous studies, suggesting that different ART treatments may not affect the probability of the reduction [58, 59].

TTTS is caused by abnormal anastomosis of blood vessels in the placenta, resulting in imbalance in blood flow from donor to recipient twin and is a major risk factor in the MC twin population [61]. Many studies documented that ART treatments can be associated with changes in some aspects of the placenta, such as morphology, structure, and growth dynamics [62, 63]. In the present study, our results showed a significantly higher TTTS rate in the TESA group. In TESA treatment, sperm retrieved from the testis instead of ejaculated. Several studies have shown that testicular sperm are of low morphological quality, have an increased incidence of chromosomal abnormalities, and exhibit different epigenetics compared to ejaculated sperm [36, 64, 65]. The main function of the sperm cell is to transmit the paternal genetic message and epigenetic information to the embryo [66]. Many genes important for placental development are expressed by paternal alleles, the male genome provides centrosomes for the first meiotic division after fertilization and is involved in the regulation of trophoblast cell proliferation, infiltration and subsequent placental proliferation [67, 68]. Sperm of different origins may have different paternally expressed genes, and have an effect on placental vascularization, and placental quality, and these placental effects may play a role in TTTS occurrence and account for the increased incidence of TTTS in our study [69]. Nevertheless, the possibility of TTTS in other groups cannot be ignored. Early abortions in the IVF group may contain some MZT pregnancies that will later develop TTTS. Meanwhile, neonatal outcomes in the TESA group were not significantly different compared with other groups, thus the phenomenon of the increased TTTS rate in the TESA group still needs to be studied with an expanded sample size. For clinical training of TTTS, early diagnosis and effective management are essential to improve pregnancy outcomes. Laser therapy is the first-line therapy for TTTS and it significantly improves survival rates and neurologic outcomes compared to amniodrainage [70].

In the neonatal outcomes MZTs conceived using different types of ART, there was no significant effect on birth weight or low weight babies in the four ART treatment groups, which was consistent with previous studies [52, 54]. He et al. found that blastocyst biopsy in the PGT group did not increase the additional risk of low birth weight when compared with the ICSI group [38]. Nan et al. investigated the neonatal outcomes in couples who had undergone ICSI using non-ejaculated sperm compared with the outcome of ICSI with ejaculated sperm and found that the baby birth weight was not significantly associated with the origin of sperm or the cause of male factor infertility [71]. In the present study, we found no significant effect on congenital malformations among four groups, as described in previous studies [35, 39]. A recent study by Wendy et al. examining maternal and neonatal outcomes after TE biopsy showed no significant differences in neonatal morbidity or birth defects between IVF with PGT and IVF without PGT [35]. Fedder et al. documented that the children born after ICSI with epididymal and testicular sperm had similar malformation rates to those born after ICSI and IVF with ejaculated sperm [39]. Our results suggested that MZTs achieved via PGT or TESA treatment did not have worse neonatal outcomes, and were reassuring for couples who may be considering the use of surgically obtained sperm or embryo biopsy. The rate of congenital malformations in each group was lower than that in the previous literature [72]. Substantial heterogeneity was previously observed in studies of obstetric risks after twin pregnancies using ART, possibly due to the differences in study populations and methods of twin pregnancy management [73, 74]. Nonetheless, congenital defects in MZTs after ART treatments need to be further investigated by enlarging the sample size.

Despite the assumption that the genome of MZTs is identical, it has been found that the probability of the discordant phenotype of congenital malformations between MC twins is approximately 9.3% [72]. Previous studies have shown that discordant malformation in MC twins is an important clinical problem affecting perinatal morbidity and mortality. There were also multiple discordant anomalies in our study including congenital cardiac anomalies and limb malformations. Helia et al. found no association between clinical risk factors and the development of discordant congenital heart defects in MZTs, suggesting that the occurrence of discordant phenotypes of congenital malformations between MZTs may be related to environmental and epigenetic mechanisms [75]. The influence of the placenta was also identified as an important factor leading to discordant congenital heart disease (CHD) with 41% of discordant CHD cases caused by placenta-related pathophysiological mechanisms [76]. A systematic review and meta-analysis suggested that monochorionic pregnancies have a twofold increased risk of structural CHD in the recipient fetus in the presence of TTTS compared with monochorionic pregnancies without TTTS [77]. Our study had three pairs of twins (all monochorionic twins) with discordant congenital cardiac anomalies, one with TTTS, one with sIUGR, and one with a weight difference greater than 20%. Due to the high incidence of TTTS in MZTs, it is important to perform fetal echocardiography and postpartum cardiac assessment in MZT pregnancies.

### Strengths and limitations

Few studies have investigated the impact of PGT and TESA treatments on pregnancy and neonatal outcomes in multiple pregnancies. Our study included a large amount of data from 2010 to 2020 covering 19,081 ART cycles for a total of 187 MZTs. Considering the relative rarity of MZTs, the number of patients was large. So far, this study is the one with the largest sample size to investigate pregnancy and neonatal outcomes of MZTs after ART treatments. Our study reported them in detail, filled part of the gap, and provides some evidence and guidance for PGT and TESA treatments. Meanwhile, the study is also of great significance to improve patient counseling before the initiation of ART treatments. Another strength is that we report on MZTs followed from the first trimester onwards after ART treatments. The majority of the previous studies only included patients from 20 to 24 weeks of gestation, and PL in the first trimester was not documented, which means that the true PL rate is underestimated [78, 79].

The limitations of this study are similar to those of other MZT studies. One of the potential limitations is the lack of fetal genetic testing for postpartum confirmation which is ideally the gold standard for the identification of MZTs [80]; however, it is expensive and temporarily not clinically achievable which may have led us to overestimate the incidence of MZTs. Meanwhile, we found MZTs by performing transvaginal ultrasonography at 6–7 weeks and, therefore, could not determine whether there was embryo loss before ultrasonography which may have reduced the incidence of MZTs. In addition, this study was limited by some data availability because the number of chorionic and amniotic sac was not routinely recorded in the ART system, and pregnancy and fetal outcomes could not be studied according to the number of amniotic sac groups. Finally, this study was retrospective, and the nature of the design inherently lends itself to treatment bias. However, because of the low incidence of MZTs, a prospective study is not feasible.

## Conclusions

In conclusion, we herein demonstrated that four ART treatments had the similar rate of MZTs. They had different impacts on pregnancy outcomes of MZTs, but had no significant effect on neonatal outcomes. The early miscarriage rate of MZTs was increased in IVF patients. Neither the cause of infertility nor the history of miscarriage was correlated with the risk of PL. MZTs in the TESA group had a higher risk of TTTS, placental effects influenced by sperm and paternally expressed genes may play a role, however, due to the small total number, studies with larger sample sizes are still needed to validate this result. Pregnancy and neonatal outcomes of MTZs after PGT treatment seem to be reassuring but the duration of the study was short, and long-term follow-up of the children is needed. Considering the serious complications associated with MZTs, the specific risk factors that increase the rate of MZT during FET cycles need further investigation.

## Electronic supplementary material

Below is the link to the electronic supplementary material.

**Additional file 1:** Supplementary Table 1. Incidence of MZT among different ART methods

**Additional file 2:** Supplementary Table 2. Pregnancy Outcome of MZT among different days of embryo

## Data Availability

The datasets used and/or analyzed during the current study are available from the corresponding author on reasonable request.

## Abbreviations

ART: Assisted reproductive technology
IVF: In-vitro fertilization
ICSI: Intracytoplasmic sperm injection
PGT: Preimplantation genetic testing
TESA: Testicular sperm aspiration
MZTs: Monozygotic twins
SET: Single embryo transfer
TTTS: Twin-to-twin transfusion syndrome
sIUGR: Selective intrauterine growth restriction
PPROM: Preterm premature rupture of membranes
GH: Gestational hypertension
GDM: Gestational diabetes mellitus
OH: Overt hypothyroidism
HT: Hashimoto’s thyroiditis
ICP: Intrahepatic cholestasis of pregnancy.
