# Supplementary material for: Pregnancy and neonatal outcomes of monozygotic twins resulting from assisted reproductive technology: a 10-year retrospective study
Source: Reprod Biol Endocrinol. 2023 Jun 2;21:51. doi: 10.1186/s12958-023-01104-7 (PMC10236834; doi:10.1186/s12958-023-01104-7)
Supplement: Supplementary file 1 — Additional file 1: Supplementary Table 1. Incidence of MZT among different ART methods [file 12958_2023_1104_MOESM1_ESM.docx]

Supplemental Table 1. Incidence of MZT among different ART methods

|  | **Total ART, n=19081** | **IVF,**  **n=8287** | **ICSI, n=3532** | **PGT, n=6223** | **TESA, n=1039** | ***P value*** |
| --- | --- | --- | --- | --- | --- | --- |
| Total MZT | 187(0.98%) | 71(0.86%) | 35(0.99%) | 66(1.06%) | 15(1.44%) | 0.259 |
| Fresh | 15/2448(0.61%) | 7(0.46%) | 6(1%) | 1(0.45%) | 1(0.90%) | 0.515 |
| Frozen | 172/16633(1.03%) | 64(0.95%) | 29(0.99%) | 65(1.08%) | 14(1.51%) | 0.430 |
| ***P value*** | **0.048*** | 0.065* | >0.999* | 0.731* | >0.999* |  |

*Note:* Data presented as n (%). * Fisher's exact test
