# Supplementary material for: Pregnancy and neonatal outcomes of monozygotic twins resulting from assisted reproductive technology: a 10-year retrospective study
Source: Reprod Biol Endocrinol. 2023 Jun 2;21:51. doi: 10.1186/s12958-023-01104-7 (PMC10236834; doi:10.1186/s12958-023-01104-7)
Supplement: Supplementary file 2 — Additional file 2: Supplementary Table 2. Pregnancy Outcome of MZT among different days of embryo [file 12958_2023_1104_MOESM2_ESM.docx]

Supplemental Table 2. Pregnancy Outcome of MZT among different days of embryo

| **Pregnancy Outcome** | **D3, n=15** | **D5, n=139** | **D6, n=33** | ***P value*** |
| --- | --- | --- | --- | --- |
| Live births | 11(73.3%) | 104(74.8%) | 22(66.7%) | 0.636 |
| Pregnancy losses | 4(26.7%) | 35(25.2%) | 11(33.3%) | 0.636 |

*Note:* Data presented as n (%).
